# The role and mechanism of fatty acid oxidation in cancer drug resistance

**DOI:** 10.1038/s41420-025-02554-1

**Published:** 2025-06-13

**Authors:** Yun Lei, Shuang Cai, Jia-Kui Zhang, Si-Qi Ding, Zhan-He Zhang, Chun-Dong Zhang, Dong-Qiu Dai, Yong-Shuang Li

**Affiliations:** 1 Department of Surgical Oncology and 8th General Surgery, The Fourth Affiliated Hospital of China Medical University, Shenyang, Liaoning Province China; 2 Department of Gastroenterology, The Fourth Affiliated Hospital of China Medical University, Shenyang, Liaoning Province China; 3 Central Laboratory, The Fourth Affiliated Hospital of China Medical University, Shenyang, China; 4 Cancer Center, The Fourth Affiliated Hospital of China Medical University, Shenyang, China

**Keywords:** Cancer metabolism, Cancer therapeutic resistance, Drug discovery

## Abstract

Cancer is a leading cause of death globally. While drug treatment is the most commonly used method for cancer therapy, it is often hampered by drug resistance. Consequently, the mechanisms of drug resistance in cancer therapy have become a focus of current research. The mechanisms underlying cancer drug resistance are complex and may involve genetic mutation, immune escape, and metabolic reprogramming, amongst others. Metabolic reprogramming is an important marker of tumor cells, and an increasing number of studies have shown that cancer drug resistance is correlated with metabolic reprogramming, especially when fatty acid oxidation (FAO) is involved. More importantly, many preclinical studies have shown that when anti-tumor drugs are combined with FAO inhibitors, cancer cell resistance to drugs can be reversed and the effectiveness of tumor therapy is enhanced. This review provides a comprehensive overview of the mechanisms by which FAO leads to cancer resistance as well as potential targets for inhibition of FAO.

## Facts

- Drug treatment is the most common form of cancer therapy and drug resistance is the main reason for the poor prognosis of cancer patients.
- Metabolic reprogramming is a common cause of cancer drug resistance, FAO has been shown to be closely associated with cancer drug resistance.
- Abnormal FAO in cancer cells can lead to drug resistance, and targeting fatty facid oxidation can reverse cancer drug resistance.

## Open questions

- What is the specific mechanism by which FAO reprogramming leads to cancer drug resistance?
- Relationship between FAO reprogramming and other metabolic reprogramming in drug-resistant cancer cells?
- Whether FAO reprogramming is associated with radiotherapy resistance in cancers?
- How to inhibit cancer drug resistance by targeting FAO?

## Introduction

Cancer is a global public health problem, with nearly 20 million new cancer cases in 2020, and the second highest number of deaths, after cardiovascular disease [1, 2]. In recent decades, due to continuous advances in medical technology and optimization of treatment options, the prognoses of most patients with malignant tumors have improved significantly, but the worldwide death toll remains high. Current treatment of tumors is generally comprehensive and individualized [3], including surgery, radiotherapy, and drug therapy. Drug therapy, in the form of chemotherapy, is the most common treatment, but it also includes endocrinotherapy, targeted therapy and immunotherapy that have emerged in recent years [4].

Many malignant tumors are effectively treated with anti-tumor drugs, which kill tumor cells that divide and grow rapidly. However, tumor treatment is often accompanied by drug resistance, when tumor cells become less responsive to treatment, which can lead to tumor recurrence or metastasis. Treatment failures are predominantly due to drug resistance [5, 6]. Drug resistance can be divided into acquired and intrinsic resistance [7, 8]. Acquired resistance refers to drug resistance that develops in cancer patients after receiving anticancer drug treatment [9]. Intrinsic resistance refers to drug resistance that is present before patients receive anticancer drugs, which is usually correlated with inherent genetic mutations in tumors [10]. Furthermore, tumor cells can develop resistance to multiple drugs with different structures and mechanisms, which is known as multidrug resistance [11]. The mechanisms underlying drug resistance are complex, and can include DNA damage repair, genetic mutation, changes in signaling pathways, abnormal transmembrane transport, immune escape, epithelial-mesenchymal transition, changes in the tumor microenvironment (TME), epigenetic changes, autophagy, and cell cycle disorders [6, 12–14]. It is worth noting that these mechanisms are not independent, but interact with each other. In practice, cancer drug resistance is often the result of a series of mechanisms working together [14, 15]. In short, the development of resistance to cancer treatment is a complex process and seriously affects the prognosis of cancer. Studying the underlying mechanisms can help overcome drug resistance and improve efficacy in cancer patients [16, 17].

Tumor metabolic reprogramming refers to the mechanism by which tumor cells promote their own proliferation and growth by changing their metabolic patterns to meet their energy needs, which not only helps the cells resist external stress, but also enables new capabilities [18, 19]. Metabolic reprogramming is common in many tumors, involving sugar, lipid, and amino acid metabolism, and is considered a major hallmark of tumors [20]. Previous studies have focused on reprogramming of glucose metabolism, but in recent years the importance of lipid metabolic reprogramming has been recognized, especially the important role of fatty acid oxidation (FAO) [21, 22]. Increasing numbers of studies have shown that FAO is associated with various diseases, especially cancer, and metabolic reprogramming of FAO is indispensable for the growth, proliferation, and metastasis of tumor cells [21, 23]. Moreover, recent studies have found that FAO can induce tumor resistance by promoting tumor cell autophagy, enhancing DNA damage repair, changing apoptosis signaling pathways, and mediating tumor cell immune evasion [24–27].

Overcoming drug resistance is a major challenge in current cancer therapy, and many studies have shown that FAO plays an important role. In this review, we first summarize the specific process of FAO and its role in tumors. The mechanism of FAO in cancer drug resistance and potential therapeutic targets to inhibit FAO are then considered and, finally, we summarize the current potential and status of FAO targeting to treat tumors.

## Overview of FAO

Metabolic reprogramming is a major feature that distinguishes tumor cells from normal cells. Uncontrolled growth and metastasis of tumor cells require metabolic reprogramming to generate sufficient energy [28]. As early as 100 years ago, Warburg discovered that even in the presence of sufficient oxygen, tumor cells tend to undergo glycolysis rather than mitochondrial oxidation. This phenomenon is known as the “Warburg effect” [29]. Since then, the metabolic reprogramming of tumor cells has gradually attracted attention. Although the ability to produce ATP is limited, aerobic glycolysis can provide tumor cells with rapid access to ATP and biosynthetic intermediates for proliferation [30]. In addition to the Warburg effect, reprogramming of amino acid and lipid metabolism also occurs, and FAO is one example of lipid metabolic reprogramming. The same dry mass of fatty acids can provide more ATP than carbohydrates or glycogen [31, 32]. Although FAO is one of the main sources of ATP production, most previous studies on cancer bioenergetics have focused on the Warburg effect, and the role of FAO in cancer has only been uncovered in recent years [30, 33].

**Fig. 1:**
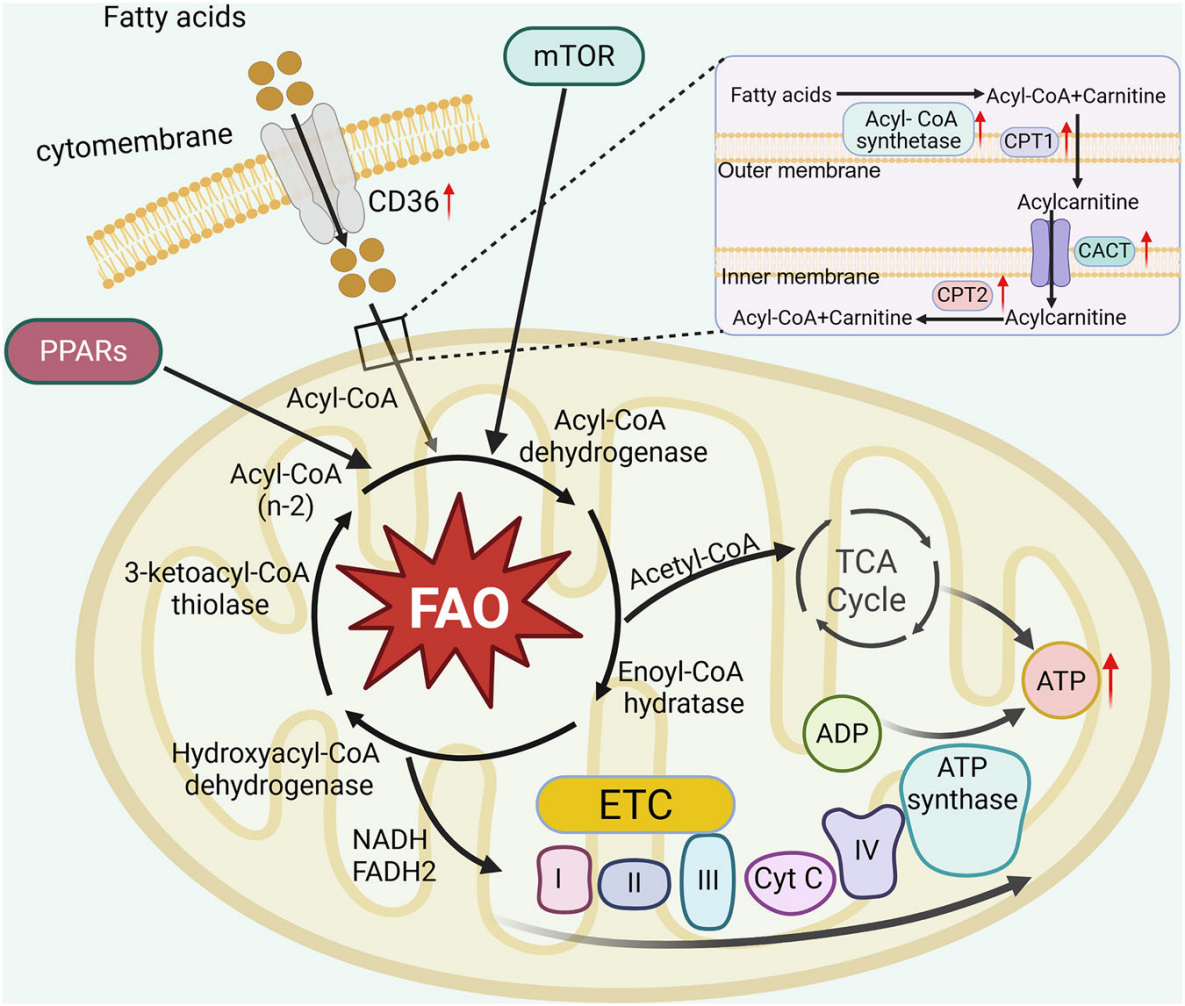
FAO in cancer. Fatty acids enter cells via fatty acid transport proteins (e.g., CD36). After entering the cell, fatty acids are first activated to acyl-CoA by fatty acyl CoA synthetase1, then converted to fatty acylcarnitine by CPT1, and finally reconverted to acyl-CoA by CACT and CPT2. Acyl-CoA is broken down into acetyl-CoA, NADH, and FADH_2_ through a four-step cycle catalyzed sequentially by acyl-CoA dehydrogenase, enoyl-CoA hydratase, hydroxyacyl-CoA dehydrogenase, and 3-ketoacyl-CoA thiolase. The resulting acetyl-CoA enters the TCA cycle to generate ATP, while NADH and FADH_2_ contribute to ATP production via the ETC. Each cycle of β-oxidation shortens the fatty acid by two carbons and generates 1 NADH and 1 FADH_2_, repeating until the fatty acid is fully oxidized to two-carbon acetyl-CoA. In cancer, aberrant overexpression of key enzymes/proteins (e.g., CD36, CPT1) drives dysregulated FAO, supporting tumor survival, proliferation, and therapy resistance. Created with BioRender.com.

FAO is a common and complex metabolic process in living organisms, through which a large amount of ATP can be produced for utilization by the body. The peroxisome proliferator activated receptors (PPARs) are the major regulator of FAO, which can promote the expression of FAO key enzymes [34, 35]. PI3K/AKT/mTOR is a key regulatory pathway for FAO, and dysregulation of this pathway in tumor cells is closely associated with abnormal FAO metabolism [36, 37]. FAO mainly takes place in cellular mitochondria, but can also occur in peroxisomes [38, 39]. Here we focus on FAO occurring in the mitochondria (Fig. 1). Fatty acids enter cells by means of fatty acid transport proteins such as CD36 [40, 41]. Upon entering the cell, fatty acids do not directly enter the mitochondria, but are first converted to acyl-coenzyme A (CoA) by fatty acyl CoA synthetase, which is the first step in the oxidation of fatty acids. At the outer mitochondrial membrane, acyl-CoA is converted to fatty acylcarnitine by carnitine palmitoyl transferase I (CPT1). Carnitine/acylcarnitine translocase (CACT), located in the inner mitochondrial membrane, translocates the acylcarnitine to the mitochondrial matrix, where carnitine palmitoyl transferase II (CPT2) on the matrix side of the inner membrane converts it to acyl-CoA [21, 42]. Acyl-CoA that reaches the mitochondrial stroma is processed in a 4-step β-oxidation cycle comprised of acyl-CoA dehydrogenase, enoyl-CoA hydratase, hydroxyacyl-CoA dehydrogenase and 3-ketoacyl-CoA thiolase, to produce acetyl-CoA, NADH and FADH_2_. The acetyl-CoA breakdown product enters the tricarboxylic acid (TCA) cycle to produce ATP, while the byproducts, NADH and FADH_2_, can participate in the electron transport chain reaction to produce ATP. In this process, with each round of β-oxidation, the fatty acid is shorted by two carbon atoms, while one NADH and one FADH_2_ molecule are produced. This process continues until the fatty acid is completely oxidized to a two-carbon acetate [31, 43, 44].

**Fig. 2:**
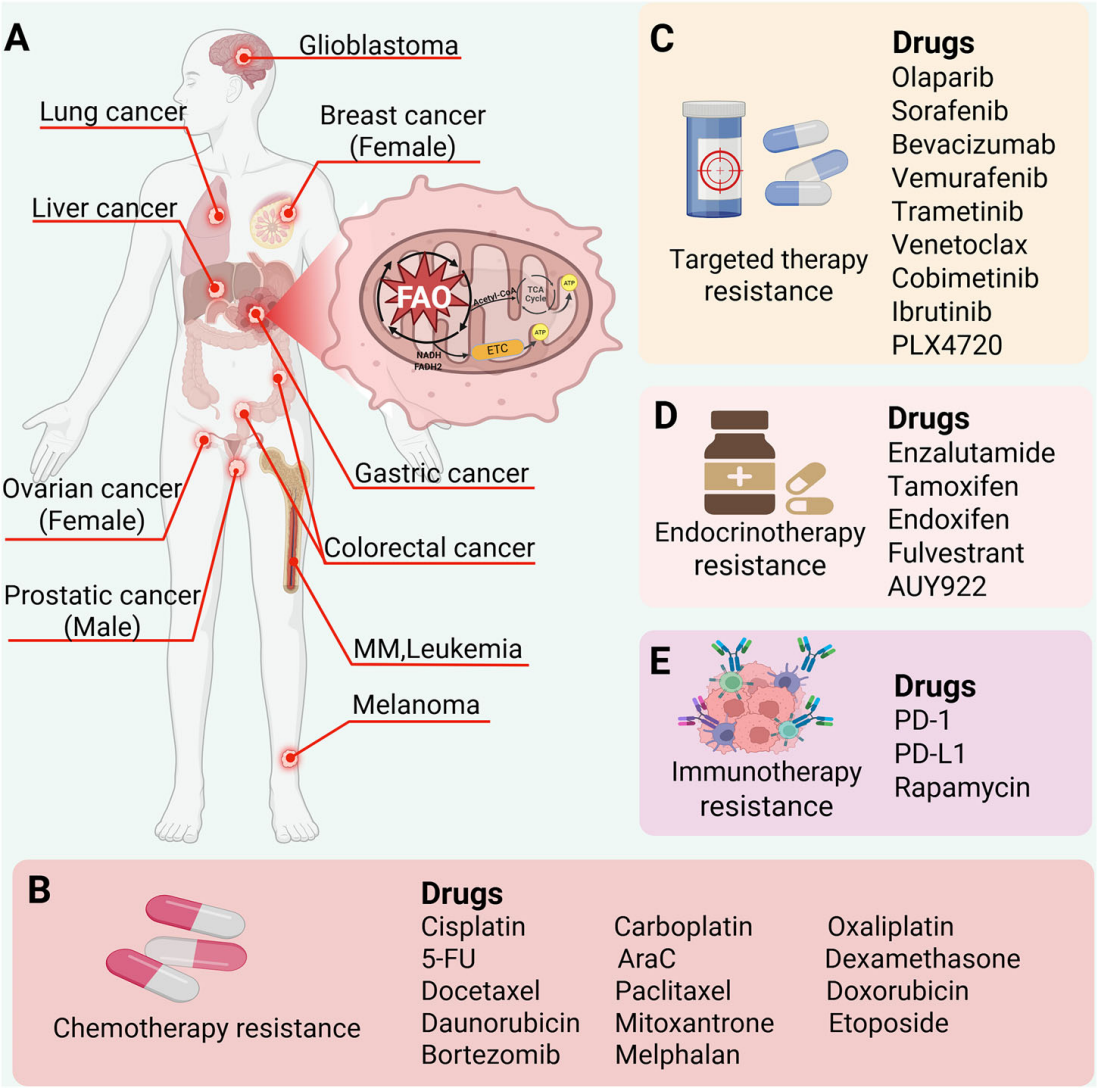
FAO and cancer drug resistance. **A** FAO can affect drug resistance in many cancers. **B** FAO affects cancer chemotherapy resistance and related drug types. **C** FAO affects cancer targeted therapy resistance and related drug types. **D** FAO affects cancer endocrinotherapy resistance and related drug types. **E** FAO affects cancer immunotherapy resistance and related drug types. Created with BioRender.com.

Under normal physiological conditions, the energy required by organs with high energy demands, such as skeletal muscle, heart, and liver, mainly comes from glucose oxidation [45]. When the glucose energy supply is limited, FAO is activated to meet energy needs, so glucose metabolism indirectly regulates FAO. Inhibition of FAO switches energy metabolism from FAO to glucose oxidation, which usually alleviates hypoxia and insulin resistance. Therefore, many FAO inhibitors can be used to treat patients with ischemic cardiomyopathy and type II diabetes [46, 47]. However, FAO is also relevant in cancer. Studies have found that many of the key mediators of FAO, such as CD36, CPT1 (including CPT1A, CPT1B and CPT1C) and CPT2 [48–50], are overexpressed in a variety of malignant tumors, and FAO has been found to be enhanced in a variety of cancers. The abnormal activation of FAO in cancers may be to meet the energy needs of rapidly proliferating tumor cells, but the role of FAO in cancers has not been fully elucidated. In recent years, studies have shown that activation of FAO is correlated with biological behaviors of tumor cells, such as survival, proliferation, invasion, metastasis, and drug resistance [21]. Especially in the case of drug resistance (Fig. 2A), studies have shown that abnormal FAO can not only directly affect drug resistance of cancer cells through metabolic reprogramming, but also by altering signal pathways, inhibiting autophagy of tumor cells and regulating the cell cycle [24, 51, 52].

## FAO and chemotherapy resistance

**Fig. 3:**
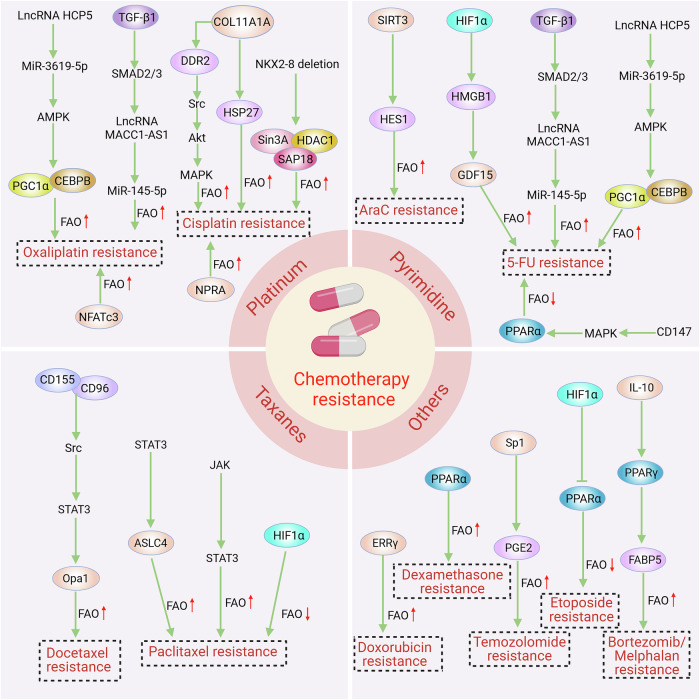
Role and mechanism of FAO in chemotherapy resistance of various cancers. Created with BioRender.com.

Although immunotherapy and targeted therapy have achieved remarkable success in cancer patients, chemotherapy remains the most commonly used drug treatment. Chemotherapy drugs can effectively kill tumor cells, but the tumor cells often develop severe drug resistance, leading to treatment failure. Increasing evidence shows that FAO activity may be correlated with cancer chemotherapy resistance (Fig. 2B) [23], Table 1 and Fig. 3 summarized the roles and mechanisms of FAO in chemotherapy resistance.

**Table 1:** Roles and mechanisms of FAO in chemotherapy resistance.

Cancer	Drug	Mechanism	Role	Ref
OC	Cisplatin	–	PR	[50]
GC	Cisplatin	The up-regulates of the NPRA strengthened the FAO	PR	[57]
GC/CRC	Oxaliplatin	NFATc3 enhances FAO by up-regulating CPT2	PR	[58]
CRC	Oxaliplatin	–	PR	[59]
GC	5-FU/Oxaliplatin	LncRNA HCP5 induces the expression of PGC1α through miR-3619-5p, promotes the activation of CPT1 by PGC1α/CEBPB complex, and enhances FAO	PR	[60]
GC	FU/Oxaliplatin	TGF-β1 increased the expression of lncRNA MACC1-AS1 by activating SMAD2/3, and lncRNA MACC1-AS1 targeted miR-145-5p to up-regulate FAO	PR	[61]
OC	Cisplatin	COL11A1 binds to DDR2 to activate the Src/Akt/AMPK pathway and enhance FAO	PR	[64]
OC	Cisplatin	COL11A1 enhances FAO by up-regulating HSP27	PR	[68, 69]
OC	Cisplatin	NKX2-8 deletion up-regulates FAO via the Sin3A/HDAC1/SAP18 transcriptional inhibition complex	PR	[72]
OC	Carboplatin	–	PR	[75]
GC	5-FU	–	PR	[80]
GC/CRC	5-FU	HIF1α signaling drives transcription of HMGB1, producing high levels of GDF15 to enhance FAO	PR	[81, 82]
CRC	5-FU	CD147 inhibits PPARα-mediated FAO by activating the MAPK pathway	PR	[86]
CML	AraC/Doxorubicin	–	PR	[90]
AML	AraC	–	PR	[91]
AML	AraC	SIRT3 SUMOylation can be achieved by up-regulating HES1 dependent FAO	PR	[94]
BC	Docetaxel	CD96 and CD155 interact to activate the Src/Stat3/Opa1 pathway and enhance FAO in cancer cells	PR	[100]
BC	Paclitaxel	Activation of the JAK/STAT3 pathway promotes up-regulation of FAO	PR	[101]
BC	Paclitaxel	Increased FAO results in acetylation of STAT3, up-regulation of ACSL4 and further increase of mitochondrial phospholipid synthesis, leading to drug resistance of cancer cells	PR	[52]
NSCLC	Paclitaxel	HIF-1α pathway activation down-regulates CPT1, thereby inhibiting FAO	PR	[102]
LC	Paclitaxel	–	PR	[103]
HCC	Doxorubicin	m6A modification promoted FAO expression by up-regulating ERRγ	PR	[107]
TNBC	Doxorubicin	–	PR	[108]
ALL	Daunorubicin	–	PR	[109]
Melanoma	Etoposide	Overexpressed HIF-1α down-regulates FAO by inhibiting PPARα	PR	[25]
MM	Bortezomib/Melphalan	IL-10 up-regulation of PPARγ/FABP5/CPT1A pathway promotes FAO up-regulation	PR	[110]
GBM	Temozolomide	Sp1 enhanced FAO by up-regulating PGE2	PR	[112]
AML	Mitoxantrone	–	PR	[113]
ALL	Dexamethasone	–	PR	[116]
CLL	Dexamethasone	Up-regulation of PPARα led to increased FAO	PR	[117]

*OC* ovarian cancer, *GC* gastric cancer, *CRC* colorectal cancer, *CML* chronic myelogenous leukemia, *AML* acute myelogenous leukemia, *BC* breast cancer, *NSCLC* non-small cell lung cancer, *LC* lung cancer, *HCC* hepatocellular carcinoma, *TNBC* triple-negative breast cancer, *MM* multiple myeloma, *GBM* glioblastoma, *ALL* acute lymphoblastic leukemia, *CLL* chronic lymphocytic leukemia, *PR* promote resistance.

### Platinum

Platinum-based chemotherapy drugs such as cisplatin, oxaliplatin, and carboplatin can induce cytotoxic DNA damage, block DNA replication and gene transcription, and lead to cell cycle arrest. They have been widely used in the clinic for treatment of malignant tumors, but resistance to platinum-based drugs is widespread [53]. The mechanisms mediating resistance to platinum drugs are complex and include weakened DNA damage effect, enhanced DNA repair, changes in signaling pathways, and metabolic reprogramming of tumor cells [54]. Resistance to platinum-based drugs has seriously affected prognosis in tumor patients. Understanding the mechanisms involved in resistance is the key to finding solutions.

Studies in recent years have shown that metabolic reprogramming of tumor cells is closely related to the development of platinum-based drug resistance, especially changes in FAO in tumor cells [50, 55]. Natriuretic peptide receptor A (NPRA) is considered to be an important receptor for atrial natriuretic peptide and has been shown to play a role in cell proliferation, apoptosis, inflammation, and tumorigenesis [56]. A recent study has shown that NPRA can enhance FAO in gastric cancer (GC) cells by protecting mitofusin 2 from protein degradation and promoting its mitochondrial localization, thereby leading to cisplatin resistance in GC cells [57]. Wang et al. found that CPT2 expression was increased in GC/colorectal cancer (CRC) cells resistant to oxaliplatin and was associated with poor prognosis. The FAO inhibitor perhexiline restored the sensitivity of resistant cells to oxaliplatin without obvious side effects. Further mechanistic study has shown that up-regulation of CPT2 is achieved through the transcription of nuclear factor of activated T cells 3 [58]. Similarly, several other studies have also found that enhancement of FAO in gastrointestinal tumors can lead to tumor cell resistance to oxaliplatin [59–61]. Collagen type XI alpha 1 (COL11A1) is a novel biomarker that is associated with chemotherapy resistance in a variety of cancers [62, 63]. A study has shown that its expression is significantly increased in cisplatin-resistant cancers [63]. A recent study found that COL11A1 activated Src-Akt-AMPK signal transduction by binding to α1β1 integrin and discoidin domain receptor 2 to up-regulate FAO, making ovarian cancer (OC) cells resistant to cisplatin [64]. Heat shock protein 27 (HSP27), a downstream effector of COL11A1, is associated with chemotherapy resistance in many types of cancer [65–67]. Evidence shows that dual inhibition of HSP27 and FAO can weaken cisplatin resistance in OC cells [68]. Further research found that HSP27 reduced levels of reactive oxygen species (ROS) in cells and mitochondria by activating the pentose phosphate pathway and glutathione (GSH) production, thus alleviating cell death induced by cisplatin and resulting in chemotherapy resistance in OC cells [69]. NKX2-8 is a homeobox-containing developmental regulator, and its down-regulation in many tumors promotes the occurrence and development of tumors [70, 71]. NKX2-8 inhibits FAO by recruiting Sin3A/HDAC1/SAP18 transcription inhibition complex, and deletion of NKX2-8 can up-regulate FAO in epithelial OC cells, resulting in their resistance to cisplatin [72]. High grade serous OC (HGSOC) is the most common and fatal epithelial OC, and most patients with HGSOC eventually develop resistance to platinum chemotherapy [73, 74]. In the platinum-resistant HGSOC cell line, expression of the FAO/oxidative phosphorylation (OXPHOS) metabolic pathway protein is increased, and inhibition of CPT1A can restore the sensitivity of HGSOC to platinum [75]. In addition, it was found that fatty acid uptake in cisplatin-resistant OC cells increased, and the increased fatty acid uptake enhanced β-oxidation by activating CPT1A, which promoted the resistance of OC cells to cisplatin. Similar phenomena also occur in many other types of cancers treated with cisplatin [pancreatic cancer, lung cancer (LC) and breast cancer (BC)], which indicates that inhibition of FAO may counter resistance to cisplatin in a variety of cancers [50].

### Pyrimidine drugs

Pyrimidine drugs are broad-spectrum anticancer drugs that act by interfering with cell metabolism. Example pyrimidines include 5 fluorouracil (5-FU), gemcitabine and cytarabine (AraC). Pyrimidine drugs are powerful anti-tumor agents, but drug resistance is the main reason for treatment failure [76, 77].

The main use of 5-FU is in the treatment of digestive tract tumors, and 5-FU resistance due to FAO has been widely reported. It was found that after mesenchymal stem cells (MSCs) were co-cultured with GC cells, the expression of long noncoding RNA HCP5 (lncRNA HCP5) in GC cells was significantly increased. Expression of PGC1α was induced by lncRNA HCP5 through miR-3619-5p, which promoted trans-activation of PGC1α/CEBPB complex on CPT1, enhanced FAO in GC cells, and led to resistance to oxaliplatin and 5-FU [60]. Similarly, when MSCs are co-cultured with GC cells, MSCs activate SMAD2/3 by secreting TGF-β1, and increase the expression of lncRNA MACC1-AS1. Overexpressed lncRNA MACC1-AS1 promotes FAO in GC cells by targeting miR-145-5p, which can also lead to resistance of GC cells to 5-FU [61]. Cancer stem cells (CSCs) have been shown to be involved in chemotherapy resistance of cancers [78, 79]. Research by Choi et al. showed that the mRNA expression level of key genes related to FAO in gastric CSCs was up-regulated, FAO was enhanced, and the use of FAO inhibitors reversed resistance of CSCs to 5-FU [80]. In addition, a study found that ROS levels increased in GC cells treated with 5-FU, which induced activation of hypoxia-inducible factor-1α (HIF-1α) signaling, driving expression of HMGB1 and resulting in more tumor-associated macrophages (TAMs) being recruited into GC. Raised levels of TAMs enhance FAO in tumor cells by producing growth differentiation factor 15 (GDF15), which leads to resistance of GC cells to 5-FU [81]. A similar mechanism of drug resistance was also found in CRC cells [82]. Increased FAO not only increases drug resistance, but has also been reported to reverse drug resistance, which may be due to the complex metabolic reprogramming of tumor cells [25]. Studies of CD147, a transmembrane glycoprotein, have shown that it is involved in malignant properties such as tumor cell metastasis, angiogenesis and drug resistance [83–85]. High expression levels of CD147 have also been found in 5-FU-resistant CRC cells. Further research found that CD147 induced resistance of CRC cells to 5-FU by regulating glucose and lipid metabolism reprogramming of tumor cells. The PI3K/AKT/mTOR pathway is activated by CD147 to up-regulate HIF-1α-mediated glycolysis, and CD147 inhibits PPARα-mediated FAO by activating the MAPK pathway, which eventually leads to drug resistance in cancer cells [86].

The main use of AraC is in the treatment of hematological diseases, where it acts by inhibiting the synthesis of deoxyribonucleic acid and interfering with cell proliferation [87, 88]. In recent years, some studies have reported a relationship between FAO and AraC resistance. Patients with chronic myelogenous leukemia (CML) and high expression of CD36 often have worse prognoses [89], higher levels of FAO in the leukemia stem cell subgroup of CML, and resistance to AraC, but the specific relationship between FAO and drug resistance and its mechanism is still unclear [90]. Similarly, acute myelogenous leukemia (AML) patients with high expression of CD36 also had worse prognoses. Chemotherapy-resistant cells showed a high OXPHOS state in AML patients, which was correlated with FAO. The use of the FAO inhibitor etomoxir ameliorated resistance of leukemia cells to AraC [91]. Sirtuin 3 is predominantly located in mitochondria, and its involvement in cellular metabolic reprogramming has been shown to correlate with chemotherapy resistance in many cancers [92, 93]. A study found that sirtuin 3 SUMOylation increased the drug resistance of AML cells to AraC by up-regulating HES1-dependent FAO. Inhibition of FAO restored the sensitivity of AML cells to chemotherapy [94].

### Taxanes

Taxanes are a class of highly effective, low toxicity, broad spectrum anticancer drugs that inhibit proliferation of cancer cells by inducing cell cycle arrest and mitotic arrest. They are widely used in the clinical treatment of malignant cancers such as BC, LC, and OC, but drug resistance cannot be ignored [95, 96]. CD96 is a newly identified immune checkpoint protein that is highly expressed in various tumor cells [97–99]. In BC, the interaction between CD96 and CD155 enhances FAO in BC cells by activating the Src-Stat3-Opa1 pathway, leading to resistance of BC cells to docetaxel [100]. In addition to docetaxel, FAO-induced paclitaxel resistance in BC has also been reported. Wang et al. found that adipocyte-derived leptin activated JAK-STAT3-CPT1B to drive FAO in breast CSCs (BCSCs), leading to paclitaxel resistance [101]. However, the mechanism by which FAO confers paclitaxel resistance in BC cells remains unknown. Subsequently, Li et al. conducted related experiments showing that elevated FAO acetylated STAT3 by increasing levels of acyl-CoA. Acetylated STAT3 up-regulates expression of acyl-coenzyme A synthase long-chain family member 4 (ACSL4), leading to increased phospholipid synthesis. The increased levels of phospholipids in the mitochondrial membrane enhanced mitochondrial integrity, thereby overcoming paclitaxel-induced tumor cell apoptosis [52]. In addition, FAO-induced paclitaxel resistance has also been reported in LC. A study found that paclitaxel resistance in non-small cell lung cancer (NSCLC) was correlated with activity of the HIF-1α pathway. HIF-1α down-regulated expression of CPT1, thereby inhibiting FAO and leading to NSCLC cell resistance [102]. In contrast, FAO expression was up-regulated in LC resistant cells, and treatment with the FAO inhibitors mercaptoacetate or etomoxir combined with paclitaxel reversed the resistance of tumor cells [103].

### Anthracyclines

Anthracyclines, including doxorubicin, epirubicin, and daunorubicin, are widely used to treat hematological malignancies and solid tumors, such as acute leukemia, lymphoma, BC, and GC. They have a broad anti-tumor spectrum and strong anti-cancer effects, but treatment resistance is also very common [104]. Estrogen receptor-related receptors (ERRs) are important mediators of multiple endocrine and metabolic signals and have been shown to be associated with BC resistance [105, 106]. A study found that N6-methyladenosine (m6A) modification significantly up-regulated ERRγ in doxorubicin-resistant hepatocellular carcinoma (HCC). Subsequently, ERRγ promoted FAO in HCC cells by up-regulating CPT1B, leading to their chemoresistance to doxorubicin [107]. It has been reported that FAO-induced doxorubicin resistance occurs not only in HCC, but also in BC. A study found that new metabolic reprogramming occurred in doxorubicin-resistant triple negative breast cancer (TNBC) cells, specifically manifested as enhanced OXPHOS and FAO. The use of FAO inhibitors can restore sensitivity to doxorubicin, but the relevant mechanism is still unclear [108]. Doxorubicin resistance due to enhanced FAO has also been reported in CML [90]. In addition to doxorubicin resistance, there have also been reports of daunorubicin resistance caused by changes in FAO. Stäubert et al. found that daunorubicin-resistant cells in acute lymphoblastic leukemia (ALL) exhibited reduced FAO, but the relationship between FAO and daunorubicin resistance is still unclear [109].

### Other drugs

Resistance-associated aberrant FAO can occur not only in cancer cells, but also in non-cancer cells in the TME, such as macrophages. Zhang et al. found that IL-10 increased lipid accumulation and FAO in macrophages through PPARγ-FABP5-CPT1A signaling in fatty acid metabolism, thereby enhancing macrophage-mediated MM resistance to bortezomib and melphalan [110]. Huang et al. found that PPARα-induced FAO regulated cell death by promoting N-alpha-acetylation of caspase-2 in melanoma mice. High expression of HIF-1α inhibited FAO by inhibiting PPARα, which led to resistance to the chemotherapeutic drug etoposide [25]. Specific protein 1 (Sp1) is overexpressed in many cancers and evidence has shown that it is closely associated with drug resistance [111]. A study found that by enhancing FAO and TCA cycles, Sp1-mediated PGE2 up-regulation increased mitochondrial ATP production, leading to glioblastoma (GBM) cell resistance to temozolomide [112]. Chemotherapy resistance is very common in AML, and lipid metabolism pathways are significantly enriched in AML-resistant cells, which leads to resistant cells with higher mitochondrial activity with enhanced OXPHOS and FAO. OXPHOS and FAO inhibitors reversed the resistance of tumor cells to mitoxantrone [113]. In addition, glucocorticoid resistance caused by FAO enhancement has also been reported. Glucocorticoids are first-line drugs for the treatment of leukemia, and glucocorticoid resistance is an important contributor to poor prognosis in leukemia patients [114, 115]. A study found that dexamethasone-resistant ALL cells underwent metabolic reprogramming from glycolysis and glutaminolysis to lipolysis, with increased FAO, and inhibition of FAO increased the cytotoxicity of dexamethasone [116]. Similarly, treatment of chronic lymphocytic leukemia (CLL) cells with dexamethasone enhanced FAO by upregulating PPARα, leading to their resistance to dexamethasone [117].

## FAO and resistance to targeted therapy

**Fig. 4:**
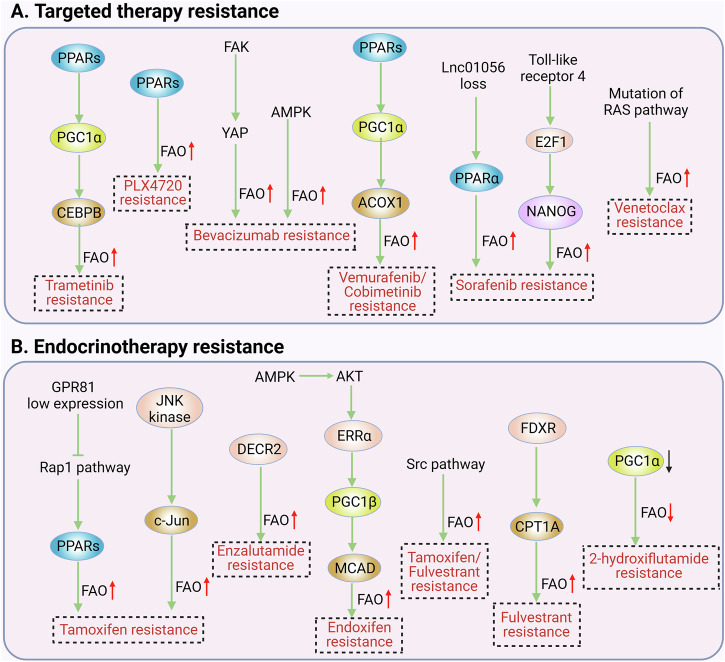
FAO in targeted therapy resistance and endocrinotherapy resistance. **A** Role and mechanism of FAO in targeted therapy resistance, **B** role and mechanism of FAO in endocrinotherapy resistance. Created with BioRender.com.

Targeted drugs, such as sorafenib, olaparib and bevacizumab, are a class of drugs that selectively act on specific tumor cell targets, thereby killing or inhibiting proliferation of tumor cells. Targeted therapies are an integral part of current cancer treatment, and despite their success, treatment-induced drug resistance remains a major cause of poor prognosis [118]. Some studies on FAO and resistance to targeted therapy have been reported recently (Fig. 2C), Table 2 and Fig. 4A summarized the roles and mechanisms of FAO in targeted therapy resistance.

**Table 2:** Roles and mechanisms of FAO in targeted therapy resistance.

Cancer	Drug	Mechanism	Role	Ref
NSCLC	Trametinib	Activation of PPARs/PGC1α/CEBPB transcription factors enhances FAO	PR	[26]
HCC	Sorafenib	Loss of lnc01056 activates PPARα, resulting in enhanced FAO	PR	[122]
HCC	Sorafenib	Toll-like receptor 4 signaling phosphorylates E2F1 to transactivate NANOG, resulting in enhanced FAO	PR	[125]
CRC	Bevacizumab	FAK/YAP pathway activation leads to FAO enhancement	PR	[126]
CRC	Bevacizumab	Up-regulation of AMPK resulted in increased FAO	PR	[127]
Melanoma	Vemurafenib	–	PR	[129]
Melanoma	Vemurafenib/Cobimetinib	Up-regulation of PPARα/PGC1-α/ACOX1 axis results in enhanced FAO	PR	[130]
Melanoma	PLX4720	Up-regulation of PPARα led to increased FAO	PR	[131]
OC	Olaparib	–	PR	[134]
AML	Venetoclax	Mutation of RAS pathway gene promotes up-regulation of FAO	PR	[135]
CLL	Ibrutinib	–	PR	[141]

*NSCLC* non-small cell lung cancer, *HCC* hepatocellular carcinoma, *CRC* colorectal cancer, *OC* ovarian cancer, *AML* acute myelogenous leukemia, *CLL* chronic lymphocytic leukemia, *PR* promote resistance.

An association between lncRNAs and cancer resistance has been demonstrated [119, 120]. A recent study found that the expression of lnc01056 is closely related to the prognosis of patients with HCC [121]. Loss of lnc01056 activates PPARα-mediated FAO, leading to resistance of HCC cells to sorafenib [122]. Tumor-initiating stem-like cells (TICs) are present in a variety of tumors, including HCC, and are involved in cancer drug resistance [123, 124]. A study found that the homeobox transcription factor NANOG inhibited mitochondrial OXPHOS and activated FAO through the TLR4/E2F1/NANOG pathway, promoting resistance of HCC TIC to sorafenib [125]. Another study reported that FAO enhancement led to cancer cell resistance to bevacizumab. A recent study identified extracellular matrix (ECM) deposition and enhanced FAO in CRC cells from patients resistant to bevacizumab. Deposition of ECM activates lipolysis in hepatic stellate cells by activating the focal adhesion kinase/yes-associated protein pathway, thereby enhancing FAO in CRC cells and leading to their resistance to bevacizumab [126]. In addition, tissue hypoxia induced by bevacizumab treatment promotes AMPK phosphorylation and enhances FAO, leading to CRC cell resistance to bevacizumab [127]. Several MAPK pathway inhibitors have been approved by the US Food and Drug Administration (FDA) for the treatment of clinical melanoma, including BRAF and MEK kinase inhibitors, which are poorly tolerated and are prone to development of resistance during treatment [128]. When melanoma cells are treated with the BRAF inhibitor vemurafenib for a long time, FAO is up-regulated, which contributes to the resistance of melanoma cells to vemurafenib [129]. In addition, a study found that peroxisomal FAO (pFAO) in BRAF-mutated melanoma persister cells was up-regulated through the PPARα-PGC1α-ACOX1 axis, and the use of pFAO inhibitors combined with BRAF/MEK inhibitors to treat melanoma delayed the emergence of resistance to targeted therapy [130]. The researchers also found that PPARα was up-regulated and FAO was enhanced in BRAF-mutated melanoma cells after MAPK inhibition therapy, leading to cancer cell resistance [131]. In addition to melanoma, a recent study reported that FAO enhancement can lead to targeted therapy resistance in LC. KRAS mutant NSCLC is highly susceptible to resistance to the MEK inhibitor trametinib. It has been reported that FAO is significantly enhanced in trametinib-resistant tumor cells, coordinately driving the OXPHOS system to meet the energy needs of tumor cells while protecting them from apoptosis. Targeting FAO can increase the sensitivity of drug-resistant tumor cells to trametinib [26]. Minimal residual disease (MRD) is closely related to cancer drug resistance [132, 133]. It has been found that advanced HGSOC MRD cells are characterized by adipocyte-like gene expression, and rely on FAO to survive and develop resistance to olapani. However, the specific mechanism leading to up-regulation of FAO is still unclear [134]. Venetoclax combined with azacytidine (ven/aza) is a very effective treatment for AML, but drug resistance is still widespread. It has been shown that resistance of AML stem cells to ven / aza occurs through the up-regulation of FAO, which occurs by a mechanism related to mutations in the RAS pathway genes [135]. Notably, the PI3K/AKT/mTOR pathway is closely related to the mitochondrial FAO [36, 136], and, more importantly, it is involved in the regulatory process of Venetoclax resistance in AML cells [137, 138]. However, the role played by the PI3K/AKT/mTOR pathway in the process of tumor drug resistance due to FAO dysregulation is still unclear. Ibrutinib is one of the main drugs used in the treatment of CLL, but drug resistance is still a problem [139, 140]. A study identified metabolic reprogramming in ibrutinib-resistant cells, which was characterized by abnormal activation of FAO. Inhibition of FAO resensitized drug-resistant cells to ibrutinib [141].

## FAO and endocrinotherapy resistance

Endocrinotherapy drugs, such as tamoxifen, fulvestrant and enzalutamide, are mainly used in the treatment of prostate cancer (PCa) and BC and act by inactivating androgen or estrogen receptors. However, there is generally resistance to endocrinotherapy drugs, which leads to treatment failure [142]. In recent years, metabolic reprogramming has been considered as an important mechanism of endocrinotherapy resistance, especially in respect of FAO (Fig. 2D), Table 3 and Fig. 4B summarized the roles and mechanisms of FAO in endocrinotherapy resistance.

**Table 3:** Roles and mechanisms of FAO in endocrinotherapy resistance.

Cancer	Drug	Mechanism	Role	Ref
BC	Tamoxifen	Low expression of GPR81 disrupts the Rap1 pathway, leading to up-regulation of PPARα and CPT1, thereby enhancing FAO	PR	[24]
BC	Tamoxifen	Transcription factor c-Jun is activated by phosphorylation mediated by JNK kinase, which in turn activates CPT1A leading to FAO enhancement	PR	[144]
BC	Endoxifen	The interaction between AMPK and AKT enhances FAO through the ERRα/PGC-1β/MCAD axis	PR	[146]
BC	Tamoxifen/Fulvestrant	FAO enhancement leads to activation of Src pathway, which leads to drug resistance in cancer cells	PR	[147]
BC	Fluvestine	FDXR enhances FAO by up-regulating CPT1A	PR	[149]
PCa	2-hydroxiflutamide	The down-regulated expression of PGC1α resulted in the inhibition of FAO	PR	[153]
PCa	Enzalutamide	–	PR	[154]
PCa	Enzalutamide	Increased expression of DECR2 led to increased FAO	PR	[155]
PCa	AUY922	–	PR	[51]

*BC* breast cancer, *PCa* prostate cancer, *PR* promote resistance.

Tamoxifen treatment can reduce cancer recurrence and mortality and is widely used as a standard first-line treatment for BC, but approximately 50% of patients eventually develop tamoxifen resistance [143]. A study found that in tamoxifen-resistant ER^+^ BC cells, the transcription factor c-Jun is activated by JNK kinase-mediated phosphorylation, which in turn activates CPT1A, leading to enhanced FAO. Inhibition of FAO can restore the sensitivity of resistant cells to tamoxifen [144]. G protein-coupled receptor 81 (GPR81) has been shown to be associated with the occurrence and development of tumors [145]. Its expression is abnormally low in tamoxifen-resistant BC cells. Low expression of GPR81 disrupts the Rap1 pathway, leading to up-regulation of PPARα and CPT1, thereby enhancing FAO and ultimately promoting resistance to tamoxifen [24]. Endoxifen resistance caused by enhanced FAO has also been reported. A study found that there is crosstalk between the AMPK and AKT pathways in BC. AMPK can promote AKT activation, while inhibition of AKT can lead to feedback activation of AMPK. The interaction between the two ultimately enhances FAO in BC cells through the ERRα/PGC-1β/MCAD/CPT-1 axis, leading to BC cell resistance to endoxifen [146]. In addition, a study found that endocrinotherapy enhances OXPHOS by up-regulating FAO in ER^+^ BC cells, thereby activating the Src pathway and leading to ER^+^ BC cell resistance to tamoxifen/fulvestrant [147]. Ferredoxin reductase (FDXR) is a mitochondrial flavin protein that is closely involved in the occurrence and development of breast cancer [148]. FDXR enhances FAO by up-regulating CPT1A, leading to ER^+^ BC resistance to fulvestrant [149].

In recent years, some studies have reported resistance of PCa endocrinotherapy caused by FAO metabolic changes. The androgen receptor inhibitor enzalutamide and other drugs are the main treatments for locally advanced and metastatic PCa, but many patients with advanced PCa die due to androgen receptor resistance [150, 151]. CSCs have the potential for self-renewal and replication in tumors, which has been shown to be correlated with cancer drug resistance [152]. One study found that PCa CSCs resistant to the androgen receptor antagonist 2-hydroxyflutamide exhibited metabolic dormancy. Expression of the key FAO enzyme, CPT1, was down-regulated and FAO was inhibited, indicating that 2-hydroxyflutamide resistance may be due to changes of FAO metabolism in CSCs [153]. Overexpression of CPT1B has been shown to be correlated with poor prognosis of PCa. In enzalutamide-resistant PCa cells, the expression of CPT1B is up-regulated, and the up-regulation of CPT1B increases the resistance of PCa cells to enzalutamide. This suggests that targeting of CPT1B may have potential for therapy of enzalutamide resistance [154]. Peroxisome 2,4-dienyl-CoA reductase 2 (DECR2) is the key enzyme in pFAO, and overexpression of DECR2 in PCa can enhance pFAO. The pFAO inhibitor thiolidazine can reverse the resistance of PCa cells to enzalutamide [155]. AUY922 is an HSP90 inhibitor that can effectively degrade androgen receptor and is widely used in the treatment of PCa [156–159]. A study has shown that FAO in PCa cells treated with AUY922 is significantly enhanced, and inhibition of FAO can increase the sensitivity of PCa cells to AUY922 [51].

## FAO and immunotherapy resistance

The successful development and application of immune checkpoint inhibitors has heralded a new era of immunotherapy for cancer treatment. However, a considerable number of cancer patients cannot benefit from it due to resistance. Metabolic reprogramming of tumor cells is the main cause of resistance, and overcoming immunotherapy resistance has become an urgent problem to be solved [160, 161]. Recently, several studies have reported immunotherapy resistance caused by metabolic reprogramming of FAO (Fig. 2E).

A study showed that CPT1A was upregulated in melanoma cells through the Wnt5a/β-catenin/PPARγ pathway, which in turn led to enhanced FAO in dendritic cells. Blocking this pathway in a melanoma model enhanced the effect of anti-PD-1 therapy [162]. In addition, FAO is enhanced when melanoma cells receive long-term BRAF inhibitor treatment, and inhibition of FAO by ranolazine can improve the efficacy of anti-PD-L1 treatment [129]. Similarly, a study has found that FAO-related gene expression is upregulated in BC and OC cells that are resistant to PD-1, and that inhibition of FAO expression can make BC/OC cells sensitive to PD-1 treatment [163]. In myeloid derived suppressor cells (MDSCs), serine/threonine kinase PIM1-mediated phosphorylation of STAT3 at the S727 site enhances STAT3 transcriptional activity and leads to increased PPARγ expression, which in turn enhances FAO expression and promotes melanoma cell resistance to PD-L1 treatment [164]. Unlike CPT1A and CPT1B, CPT1C mainly plays a role in the brain, but a recent study found that it is also highly expressed in malignant cancers such as LC [165, 166]. CPT1C is over-expressed under AMPK regulation when tumor cells are hypoxic, which enhances FAO and leads to resistance of colon cancer cells to rapamycin [48]. Because immunotherapy drugs have only been used in the clinic for a short time, there are few studies on immunotherapy resistance caused by FAO metabolic reprogramming. However, numerous studies show that FAO is involved in immunotherapy [167–169]. More studies are needed to clarify the relationship between FAO and immunotherapy resistance.

## FAO is a potential target for reversing cancer drug resistance

Obviously, tumor cells are different from normal cells, and the activities of tumor cells require FAO energy [170, 171]. Therefore, based on the difference in the demand for FAO between tumor cells and normal cells, tumors can be treated by targeting FAO, and the side effects on normal cells should be minimal. Drugs targeting FAO have been shown to be effective and safe for clinical use [47, 172]. Some of these drugs have been used in the clinical treatment of angina pectoris in the USA, Australia and other regions. These drugs, which include perhexiline, trimetazidine and ranolazine [173–175], mainly improve glucose oxidation in cardiomyocytes by inhibiting FAO. Although the clinical use of FAO inhibitors in oncology is not yet widespread, several preclinical studies have demonstrated that FAO inhibitors have great potential for reversing cancer drug resistance (Table 4). For example, the FAO inhibitor etomoxir reversed cisplatin resistance in cisplatin-resistant cells from OC and GC [57, 64], perhexiline reversed AUY922 resistance in PCa cells [51], and thiolidazine reversed enzalutamide resistance in PCa cells [155]. In addition, many studies have shown that combination therapy with FAO inhibitors can enhance the therapeutic effect of anti-tumor drugs [58, 129, 176, 177]. A study found that alisertib combined with trametinib treatment in BRAF mutant melanoma patient-derived drug resistance models can alter metabolic reprogramming and enhance FAO. Etomoxir combined with alisertib and trametinib enhanced the therapeutic effect and significantly prolonged the overall survival of mice [178]. Aurora kinase A (AURKA) is a therapeutic target for GBM, and combination of an AURKA inhibitor and an FAO inhibitor prolonged total survival time in a GBM patient-derived xenograft model [179]. Taken together, targeting FAO may be a potential target for reversing cancer drug resistance.

**Table 4:** Application of common FAO inhibitors in cancer drug resistance.

Inhibitor	Target	Cancer	Drug	Ref
Etomoxir	CPT1	BC, CRC, GC, CLL	Tamoxifen, Oxaliplatin, Bevacizumab, Ibrutinib	[24, 59, 126, 141, 147]
Perhexiline	CPT1/CPT2	OC, PCa	AUY922, Cisplatin, Carboplatin	[51, 72, 75]
Ranolazine	3-KAT of TFP	Melanoma	Vemurafenib	[129]
Mercaptoacetate	Acyl-CoA dehydrogenase	LC	Paclitaxel	[103]
Thioridazine	–	Melanoma, PCa	Enzalutamide, Vemurafenib, Cobimetinib	[130, 155]

*CPT* carnitine palmitoyl transferase, *TFP* the trifunctional protein (acyl-CoA dehydrogenase/enoyl-CoA hydratase/3-KAT), *BC* breast cancer, *CRC* colorectal cancer, *GC* gastric cancer, CLL chronic lymphocytic leukemia, OC ovarian cancer, PCa prostate cancer, LC lung cancer.

It is worth noting that although studies have shown that FAO inhibitors alone can inhibit the growth of tumor cells and reverse drug resistance, these drugs usually cannot completely eliminate tumor cells [180]. Therefore, FAO inhibitors are usually used in combination with anti-tumor drugs for tumor treatment. At present, combined therapy is advocated for cancer treatment, especially drug combination therapy [4]. A large amount of evidence shows that compared with single drug therapy, multi-drug combination therapy can enhance efficacy and reduce toxicity, achieve the same or better therapeutic effect without increasing drug dosage, and effectively reduce the development of drug resistance [181]. Therefore, the addition of FAO inhibitors to conventional antitumor drug regimens can improve therapeutic efficacy and reverse cancer drug resistance, which has great potential in tumor therapy [179].

## Future perspectives and conclusion

It has been a century since Otto Warburg put forward the viewpoint that metabolic reprogramming occurs in tumor cells, and the role of metabolic reprogramming in tumor occurrence and development has been fully studied [29]. In recent years, the influence of FAO on cancer resistance has been demonstrated by a large number of studies, especially in the field of chemotherapy resistance. However, evidence connecting FAO and tumor immunotherapy resistance is still lacking. Many studies have shown that targeting FAO can enhance the response of tumor cells to immunotherapy [27, 182, 183]. With the wide application of immunosuppressants in clinical tumor treatment, the relationship between FAO and drug resistance in immunotherapy will be gradually revealed. We believe that studying the role of FAO metabolic reprogramming in immunotherapy will become the focus of future work in the field of cancer. In addition to drug resistance, some studies have shown that changes in FAO will also affect radiotherapy resistance in tumor patients. It has been found that up-regulation of FAO can lead to radiotherapy resistance in both BC and nasopharyngeal carcinoma, and targeting CPT1A can eliminate this resistance [184–186]. Therefore, the inhibition of FAO may have potential for reversal of radiotherapy resistance. More research will be needed to confirm this view in the future.

The PI3K/AKT/mTOR signaling pathway is a central hub of cellular metabolic regulation, playing a key role in lipid metabolic reprogramming [36]. This pathway dynamically regulates the balance between lipid synthesis and FAO, which is often disrupted in malignant tumors, leading to the ability of tumor cells to adapt to microenvironmental stress and escape treatment [37, 187]. In normal cells, the PI3K/AKT/mTOR pathway tends to inhibit FAO and promote lipid synthesis to store energy and avoid excessive mitochondrial oxidative stress. However, in cancer cells, dysregulation of the PI3K/AKT/mTOR pathway drives reprogramming of lipid metabolism, which is manifested by abnormal enhancement of FAO, which not only provides ATP for tumors, but also inhibits apoptosis and regulates redox homeostasis, which ultimately leads to the development of tumor drug resistance [36, 188, 189]. Therefore, targeting the PI3K/AKT/mTOR signaling pathway may be the key to reversing tumor drug resistance.

In overweight and obese states, the body produces more free fatty acids (FFAs) [190]. These FFAs can remodel the TME and promote tumor progression through mechanisms such as activating signaling pathways, promoting the formation of immune microenvironment, and regulating metabolic reprogramming [191–194]. In addition, these FFAs can be efficiently taken up by tumor cells via transporter proteins such as CD36, leading to over-activation of mitochondrial FAO, which provides the energy base for chemotherapy resistance [195]. During nutritional adequacy, FFAs are converted to triglycerides and stored in lipid droplets; whereas, under chemotherapeutic stress, the lipid droplets are rapidly broken down to release FFAs, which promotes tumor drug resistance through enhanced FAO [196]. PPARγ has been shown to be closely associated with cancer progression [197]. PPARγ, as a key regulator of FAO, is required for adipocyte differentiation [198, 199], especially under obese conditions [200]. and adipocyte de-differentiation may be a process of adaptation to pathophysiological stressful environments, which has been shown to promote tumor progression [191]. This suggests that the regulatory effect of PPARγ on adipocyte differentiation under obese conditions may be associated with cancer progression. In conclusion, several studies have demonstrated that FFAs are more likely to lead to tumor resistance by promoting FAO under obese conditions, and that PPARγ, as a common regulator of FAO and adipocytes, has great potential in tumor therapy.

Reprogramming of lipid metabolism is one of the important features of malignant tumors, including fatty acid synthesis in addition to FAO. Fatty acid synthesis, as an upstream supplier of lipid metabolism, not only provides cancer cells with lipids for membrane construction and energy storage, but also regulates the substrate supply of FAO through dynamic homeostasis, which together maintains the malignant phenotype of the tumor [201]. Normal cells are mainly dependent on exogenous lipid uptake, whereas cancer cells continue to activate the fatty acid synthesis pathway even in lipid-rich environments. Overexpression of fatty acid synthase (FASN) has been found in a variety of solid tumors, including breast cancer and prostate cancers, and its levels correlate significantly with tumor stage, metastasis and poor prognosis [202, 203]. Fatty acid synthesis facilitates cellular energy storage, maintains redox homeostasis, participates in signaling pathway regulation, and meets the demands of cell membrane synthesis. Recent study has revealed that targeting fatty acid synthesis can effectively inhibit tumor progression and reverse drug resistance, providing a new direction for metabolic intervention [204]. Inhibitors targeting key enzymes of fatty acid synthesis, such as ACC inhibitors and FASN inhibitors, have already achieved significant therapeutic efficacy in a variety of tumors [205]. Therefore, targeting fatty acid synthesis in combination with FAO is a very promising direction in the reversal of drug resistance in tumors.

Although some FAO inhibitors, such as etomoxir, have shown great potential in preclinical experiments, their clinical transformation has been hampered by high toxicity, dose tolerance and poor specificity [206]. Therefore, in order to achieve precise targeting of FAO in the field of cancer, it is necessary to study FAO inhibitors with higher specificity and lower toxicity in the future [207]. In addition, although most current studies support the view that up-regulation of FAO promotes cancer drug resistance, some studies have reached the opposite conclusion [25, 86, 102, 109, 153]. This may be because metabolic reprogramming of the tumor is an extremely complicated process. Different tumors are subject to different metabolic reprogramming, and the same tumor may also be susceptible to many kinds of metabolic reprogramming. As a consequence, the same metabolic reprogramming may lead to different biological behaviors in different tumors, and biological behaviors in the same tumor may also differ due to alternative metabolic reprogramming [25]. More research is needed in the future to clarify the role of FAO in cancer drug resistance. The emergence of new technologies, such as tumor metabolism determination technology and single cell sequencing, will be beneficial to the study of metabolic reprogramming in tumor cells and contribute to the early conquest of cancer.

In conclusion, a large number of studies have shown that metabolic reprogramming of FAO can affect cancer drug resistance, and that targeting FAO has potential for reversing cancer drug resistance. This approach has great potential in tumor therapy, but there is still a long way to go to achieve clinical application.
